# Comparison of the Effects of Isokinetic, Isometric, and Isotonic Exercises on Knee Osteoarthritis Using Ultrasound

**DOI:** 10.7759/cureus.28324

**Published:** 2022-08-23

**Authors:** Ayşe Bahşi, Özlem Altındağ, Mazlum Serdar Akaltun, Ali Aydeniz, Ender Ersin Avcı, Ali Gür

**Affiliations:** 1 Algology, Çukurova University, Adana, TUR; 2 Physical Medicine and Rehabilitation, Gaziantep University, Gaziantep, TUR; 3 Physiotherapy and Rehabilitation, Marmara University, Istanbul, TUR

**Keywords:** exercise, femoral cartilage thickness, ultrasound (u/s), osteoarthritis (oa), knee

## Abstract

Introduction: This study aims to investigate the effects of different types of exercise on pain, functional capacity, muscle strength measured by an isokinetic dynamometer, and femoral cartilage (FC) thickness measured using ultrasound in patients with knee osteoarthritis (KOA).

Methods: Sixty patients were randomized to isokinetic, isometric, and isotonic exercise groups, and exercise programs were completed five days a week over three weeks. The FC thickness for each knee was evaluated in medial, intercondylar, and lateral areas using ultrasound, and muscle strength was assessed by an isokinetic dynamometer. Activity levels were also measured using the Western Ontario and McMaster Universities Osteoarthritis Index (WOMAC). At three weeks from baseline, follow-up clinical measurements of knee muscle strength and FC thickness were performed.

Results: A significant decrease was observed in all three groups in post-treatment visual analog scale (VAS) pain scores. In terms of evaluation of WOMAC scores, no significant difference was observed between the groups. In the isokinetic group, a significant increase was detected in extensor and flexor muscle strength measurements at the angular velocities of 60º/s and 180º/s. In the isotonic group, a statistically significant increase was detected in peak torque values of extensor and flexor muscles at 60º/s in the left knee only. Regarding FC thickness, a significant increase was found in the intercondylar area and the lateral condyle measurements of the left knee in the isokinetic group and the intercondylar area measurements of the right knee in the isometric group. In the isokinetic group, a statistically significant decrease was detected in the medial condyle of the right knee. It was observed that isokinetic exercise ended up with a significant increase in FC thickness in the intercondylar area and the lateral condyle of the left knee and a significant decrease in the medial condyle of the right knee.

Conclusion: Isokinetic exercise is functionally superior to isometric and isotonic exercise, providing more significant improvements in muscle strength measurements and FC thickness. Further research is needed to determine the interactions among therapeutic exercises in patients with KOA that impact knee cartilage quantities.

## Introduction

Osteoarthritis is a progressive, degenerative, non-inflammatory disease, and the prevalence of which increases with advanced age. It is characterized by cartilage destruction, subchondral sclerosis, and osteophyte formation, and in deteriorated cases, osteocytes, especially in weight-bearing joints [1,2]. Osteoarthritis is one of the major causes of physical disability. Besides, it is the most common indication for knee and hip replacements [2,3]. Although it may affect various joints in the body, it is most common in the knees. The American College of Rheumatology (ACR) has established clinical and radiological diagnosis criteria for knee osteoarthritis (KOA) [4].

The aims of KOA treatment are patient education, pain management, improving joint function, and reducing limitations. Exercise, one of the cornerstones of general treatment, is among the recommended non-pharmacological treatments in ACR’s treatment guides published in 2012 [5]. The most common exercise therapy methods include a joint range of motion, stretching, strengthening, and aerobic exercises. Strengthening exercises such as isometric, isotonic, and isokinetic exercises may also be performed. A great number of studies have reported that exercises relieve pain, increase functional capacity and muscle strength, and reduce medications for patients with KOA [6-9].

Ultrasound (US) is a non-invasive, convenient, and inexpensive technique to evaluate KOA. It can be performed comfortably at the bedside, and it does not involve ionizing radiation [10,11]. The US of the knee is a reliable method in the evaluation of the periarticular soft tissue and intraarticular structures, and also the morphological features of the soft tissue that conventional radiography is unable to define [12-16].

Although exercise therapy is frequently used in patients with KOA, there are a limited number of studies on the effects of different exercise treatments on femoral cartilage thickness [10,17,18]. This study aims to investigate the effects of different types of exercise on the pain, functional capacity, the thickness of the femoral cartilage measured using ultrasound, and muscle strength measured with an isokinetic dynamometer in patients with KOA, and thereby contribute to the creation of an ideal treatment program.

## Materials and methods

This study was conducted in patients with radiological stage two or three primary KOA, according to OA diagnosis criteria of ACR. Sixty volunteer patients (56 females, four males) between the ages of 45 and 65 were admitted to the outpatient clinic of the Department of Physical Medicine and Rehabilitation at the Gaziantep University, School of Medicine between March 2015 and January 2016. Approval for this study was obtained from the Gaziantep University Clinical Trials Ethics Committee (date: 09.03.2015, decision no: 2015/88). Written and verbal information was given to the patients regarding the treatment to be undertaken and preventive methods in accordance with the Ethics Committee’s requirements.

The exclusion criteria include those needing support when walking, pregnancy, history of knee surgery, inflammatory arthritis, tumor, infection, psychiatric disorder, or hemorrhagic diathesis, the presence of limited range of knee and ankle joint movement, having a cardiac pacemaker, doing regular exercise, the presence of reflecting pain from the lower back or the hip.

The patients diagnosed with osteoarthritis of the knee were evaluated and randomized into three groups: isometric, isotonic, and isokinetic exercise. The demographic data (age, weight, height, body mass index (BMI)) of the patients were recorded. Pain of the patients were assessed using the visual analog scale (VAS) [19]. Activity levels of the patients was also measured by Western Ontario and McMaster Universities Arthritis Index (WOMAC) [20]. The validity and reliability of the WOMAC questionnaire for Turkish version was conducted by Tüzün et al. [21]. Pre and post treatment VAS and WOMAC scores were recorded.

Isokinetic evaluation

Isokinetic muscle strength evaluation was performed by the isokinetic dynamometer HUMAC 2009, v.9.7.1 (Computer Sports Medicine Inc., Stoughton, MA, USA). The pre and post-treatment muscle strengths of all patients were measured and recorded (Video 1). The patients were seated in an upright position, with the backrest at 85°. The lever arm pad was secured just proximal of the medial malleus. The lateral femoral epicondyle was used as a bone landmark for matching the knee’s anatomical axis of rotation with the mechanical axis of the dynamometer [22]. Angular velocities of 60°/s and 180°/s were carried out during the test phase. Patients performed five submaximal repetitions at the angular velocities of 60°/s and 180°/s before starting the test. Five repetitions were made at the maximum strength at 60°/s for the strength test. Five repetitions were made at the maximum strength at 180°/s for the endurance test. The patients took a break of 20 seconds between the changes in angular velocity.

**Video 1:** Isokinetic muscle strength evaluation

Ultrasonographic measurements

The patients' pre and post-treatment ultrasound measurements were conducted at the medial, intercondylar and lateral aspects by the same investigator blinded to the treatment groups using a 2-13 MHz linear probe (Chison Medical Imaging Co. Ltd, New District, Wuxi, China). To measure the femoral cartilage thickness, the patients were placed on the examination table in the supine position, with the knees in the maximum flexion in a comfortable position, while the probe was placed axially on the suprapatellar region. An anechoic structure was observed between the bone cortex and the suprapatellar fat pad in the distal femoral cartilage. The femoral cartilage thickness was measured at three points on both knees at the lateral condyle, intercondylar area and medial condyle levels pre-treatment and post treatment.

Exercise program

The patients in the first group were included in an isometric exercise program. The knees were extended straight in the anatomical position and patients exercised for 20 minutes, with a maximal effort of 10 seconds, five days a week for three weeks under the supervision of the investigator.

The patients in the second group were included in an isotonic exercise program. They performed weightlifting exercises with weights of 1.5 kg against gravity with 90 repetitions, five days a week for three weeks, under the supervision of the investigator.

The patients in the third group were included in an isokinetic exercise program. They performed concentric-concentric isokinetic flexion and extension exercises at the velocity range of 60°/s and 180°/s, at 30°/s intervals with 10 repetitions at each velocity, five days a week for three weeks. They took a rest of 20 seconds at each angular velocity, and of five minutes between each knee. All patients in the groups were evaluated before and after the exercise therapy.

Statistical analysis

The statistical analyses of the data were performed using Statistical Package for Social Sciences (SPSS) version 22 (IBM Corp., Armonk, NY, USA). The normal distribution of the data was tested using the Shapiro-Wilk test. The analysis of variance (ANOVA) and least significant difference (LSD) tests were used to compare the numerical data with normal distribution between the three groups, whereas the Kruskal-Wallis test and Dunn’s multiple comparison test were used for the comparison of data with non-normal distribution. The Wilcoxon test was used to compare the measurements obtained from the numerical variables at two different time points. The relationship between categorical variables was determined using the chi-square test. The p-values below 0.05 were considered statistically significant.

## Results

Sixty patients (56 females, four males) diagnosed with primary osteoarthritis of the knee were included in the study. The mean age of the patients was 54.5±6.25 years, and their mean BMI was 33.4±5.6 kg/m^2^. No significant difference was observed between the groups in terms of the mean values of age, weight, height, and BMI (Table 1).

**Table 1:** Mean values of the age, height, weight, and BMI of the patients Twenty patients were evaluated in all three groups. †: Mean ± standard deviation, BMI: Body mass index

Parameter	Isometric Exercise ^†^	Isotonic Exercise ^†^	Isokinetic Exercise ^†^	p-value
Age	56.25±6.59	54.55±6.11	52.70±5.84	0.202
Weight	88.65±17.90	89.45±13.36	80.65±12.62	0.121
Height	1.61±0.06	1.59±0.83	1.59±0.05	0.450
BMI	33.8±5.92	34.85±5.35	31.6±5.43	0.180

When the pain intensity was assessed on the VAS in each of the three groups, a significant decrease was observed in the post-exercise values compared with the pre-exercise values (p<0.05) (Table 2). The comparison of the exercise groups did not reveal a statistically significant difference between the groups in terms of the decrease in pain intensity (p>0.05).

**Table 2:** Comparison of the VAS, WOMAC, and peak torque values pre and post-treatment in the three groups ^†^: Mean ± standard deviation, *: Significant difference (p<0.05), Pre: Pre-treatment, Post: Post-treatment, PT: Peak torque values, R: Right, L: Left, Ex60pt: Extensor peak torque values at 60°/s, Ex180pt: Extensor peak torque values at 180°/s, Flx60pt: Flexor peak torque values at 60°/s, Flx180pt: Flexor peak torque values at 180°/s, WOMAC: Western Ontario and McMaster Universities Arthritis Index, VAS: Visual analog scale

	Isometric Exercise			Isotonic Exercise			Isokinetic Exercise		
	Pre ^†^	Post ^†^	p	Pre ^†^	Post ^†^	p	Pre ^†^	Post ^†^	p
VAS pain	8.70±1.59	5.40±2.90	0.001*	8.30±1.89	4.85±3.21	0.001*	8.05±1.93	3.75±2.67	0.001*
WOMAC pain	11.90±4.14	8.75±5.70	0.003*	11.65±3.61	7.50±4.66	0.006*	12.90±3.68	6.95±4.66	0.001*
WOMAC stiffness	4.60±2.45	2.75±2.46	0.001*	4.10±2.26	2.90±2.33	0.022*	4.95±2.37	2.45±2.43	0.001*
WOMAC physical function	46.10±14.49	33.55±17.59	0.001*	46.10±12.11	28.55±16.75	0.001*	43.65±10.81	24.75±16.45	0.001*
WOMAC total	62.60±20.15	44.95±24.86	0.001*	61.85±16.37	38.95±22.54	0.001*	61.45±14.65	34.10±22.26	0.001*
Ex60pt (R)	54.75±24.04	55.60±31.53	0.614	60.20±25.57	69.85±30.79	0.455	59.15±18.61	70.65±19.69	0.014*
Flx60pt (R)	20.05±7.74	21.50±8.31	0.432	25.15±10.50	30.90±16.70	0.055	29.15±10.57	37.15±11.37	0.002*
Ex180pt (R)	34.00±15.49	32.10±16.11	0.169	36.75±18.71	42.30±17.95	0.433	30.85±13.88	39.00±11.99	0.007*
Flx180pt (R)	11.75±6.30	13.25±5.99	0.213	18.50±8.14	19.00±9.99	0.896	15.95±8.33	21.15±5.15	0.024*
Ex60pt (L)	54.55±22.31	54.50±20.05	0.990	51.80±21.50	68.45±24.54	0.021*	57.55±21.55	65.60±21.74	0.004*
Flx60pt (L)	23.05±8.64	25.30±7.87	0.254	24.15±12.49	31.25±14.01	0.044*	30.55±13.13	36.60±13.57	0.033*
Ex180pt (L)	33.95±11.32	33.00±14.00	0.586	34.00±16.97	40.45±17.48	0.144	27.35±11.27	40.95±13.44	0.001*
Flx180pt (L)	14.75±7.35	15.55±7.15	0.936	16.70±10.11	20.20±9.27	0.168	16.70±8.08	22.95±9.06	0.005*

When the pain intensity was assessed on the WOMAC in each of the three groups, a statistically significant decrease was observed in the post-exercise therapy values compared with the pre-exercise therapy values (p<0.05) (as seen above in Table 2). In terms of evaluation of pain intensity, no significant difference was observed between the groups (p>0.05)

There was no statistically significant increase detected in either the extensor or flexor muscles' peak torque values at the angular velocities of 60º/s and 180º/s in both knees in the isometric exercise group compared with the pre-exercise therapy values (p>0.05) (as seen above in Table 2). No significant difference was detected in either the extensor or flexor muscles' peak torque values at the angular velocities of 60º/s and 180º/s in both knees in the isometric exercise group compared with the pre-exercise therapy values (p>0.05) (as seen above in Table 2).

In the isotonic exercise group, a statistically significant increase was detected in the peak torque values of the extensor and flexor muscles at 60º/s in the left knee (p<0.05). No statistically significant increase was found in the peak torque values of the extensor and flexor muscles at 60˚/s and 180˚/s in the right knee and at 180˚/s in the left knee (p>0.05) (as seen above in Table 2). No significant difference was observed in terms of peak torque values of the extensor and flexor muscles at 60˚/s and 180˚/s in the right knee and at 180˚/s in the left knee (p>0.05) (as seen above in Table 2).

A statistically significant difference was detected in the pre- and post-exercise isokinetic peak torque values of the extensor and flexor muscles at 60º/s and 180º/s in the right and the left knees of the patients in the isokinetic exercise group (p<0.05) (as seen above in Table 2).

While in the femoral cartilage thickness assessment, a statistically significant difference was detected between the pre- and post-treatment measurements of the intercondylar area of the right knee in the isometric exercise group (p<0.05), no statistically significant increase was detected in either of the measurements of the medial and lateral measurements of the right knee or the medial, intercondylar and lateral measurements of the left knee (p>0.05). No significant difference was detected in the ultrasonography measurements of the right and the left knees in the isotonic exercise group (p˃0.05). In the isokinetic exercise group, while a significant decrease was observed in the medial condyle of the right knee (p<0.05), a significant increase was observed in the lateral and intercondylar cartilage thicknesses of the left knee (p<0.05) (Table 3).

**Table 3:** Comparison of the medial condyle, intercondylar area, and lateral condyle measurements pre and post-treatment R: Right knee, L: Left knee, Pre: Pre-treatment, Post: Post-treatment, MC: Medial condyle, IA: Intercondylar area, LC: Lateral condyle, ^†:^ Mean ± standard deviation, *: Significant difference (p<0.05), ^α^: Centimeter

	n	MC (R) ^α^			IA (R) ^α^			LC (R) ^α^			MC (L) ^α^			IA (L) ^α^			LC (L) ^α^		
		Pre ^†^	Post ^†^	p	Pre ^†^	Post ^†^	p	Pre ^†^	Post ^†^	p	Pre ^†^	Post ^†^	p	Pre ^†^	Post ^†^	p	Pre ^†^	Post ^†^	p
Isometric Exercise	20	0.25±0.05	0.26±0.06	0.125	0.26±0.09	0.28±0.07	0.023*	0.26±0.05	0.26±0.05	0.570	0.24±0.06	0.23±0.06	0.820	0.24±0.06	0.26±0.06	0.069	0.26±0.06	0.26±0.05	0.710
Isotonic Exercise	20	0.21±0.06	0.22±0.06	0.385	0.23±0.05	0.24±0.06	0.458	0.25±0.05	0.26±0.06	0.558	0.24±0.06	0.23±0.05	0.121	0.25±0.06	0.25±0.05	0.913	0.25±0.04	0.26±0.05	0.141
Isokinetic Exercise	20	0.25±0.06	0.24±0.05	0.042*	0.25±0.06	0.26±0.06	0.694	0.25±0.06	0.24±0.05	0.235	0.24±0.05	0.25±0.05	0.550	0.23±0.05	0.26±0.06	0.020*	0.24±0.05	0.26±0.05	0.041*

## Discussion

Osteoarthritis is a progressive, degenerative, non-inflammatory disease the prevalence of which increases at an advanced age. It has been reported that exercise can relieve pain and improve physical function in patients with KOA [6,23]. However, information on the type or intensity, duration, and long-term effects of exercise for KOA is still unclear. There is a variety of studies in the literature investigating the effects of isometric, isotonic, and isokinetic exercises on pain, functionality, and walk speed in patients with KOA [10,17,18].

There are a limited number of studies comparing cartilage thickness in different exercise groups [17]. This study aims to clarify an important gap in the literature. The present study compares femoral cartilage thickness among isometric, isotonic, and isokinetic exercises before and after they are performed.

In previous studies, significant changes were observed in patients with KOA who exercised to reduce pain levels and increase functional capacity compared to the control group [8,24-27]. It is considered that exercise has a positive effect on pain and functional capacity. Similarly, it was demonstrated in the present study that there was a remarkable improvement in pain state and functional capacity. Additionally, in previous studies, it was reported that significant changes were obtained in muscle strength, peak torque levels, and walking speed in exercise groups compared to control groups [8,24,26]. In the present study, we observed a significant improvement in the isokinetic group for all parameters. In our opinion, this data was objective dues to the usage of the isokinetic test and treatment device. In addition, Gür et al. [8] reported that in patients with knee osteoarthritis who performed concentric and concentric-isokinetic exercises, there was a decrease in pain levels and an increase in the functional capacity, the strength of the cross-section of the muscles, and the peak torque in both exercise groups compared to the control group. On the other hand, although the positive effects of exercise on symptoms of disease in patients with KOA have been proved, the effects of exercise on femoral cartilage thickness have not been well studied.

Eyigor et al. [24] stated that in patients with KOA who received isokinetic exercises and progressive resistance exercises, significant improvement was observed in the pain state, functional capacity, walking duration, and muscle strength of both groups. At the same time, they did not find a significant difference between the groups in terms of these results [24]. Similarly, a decrease in pain intensity and an increase in functional capacity were detected in the present study in all three groups. There was no significant difference between the groups in terms of pain and increase in functional capacity. As for the muscle strength evaluation, a statistically significant increase was detected in the muscle strength evaluated by the peak torque values of the extensor and flexor muscles at 60°/s and 180°/s in the right and left knees in the isokinetic exercise group. In the isotonic exercise group, a statistically significant increase was detected in the peak torque value of the extensor and flexor muscles at 60º/s in the left knee only. This study shows that isokinetic exercises increase extensor and flexor muscle strengths more than isometric and isotonic exercises.

Huang et al. [26] applied isokinetic, isotonic, and isometric strengthening exercise therapy to patients with bilateral KOA, with a fourth group assigned as the control group. They found a decrease in pain, decrease in walking speed, and disability in all groups. The greatest decrease in pain was achieved in the isotonic exercise group, an increase was detected in muscle strength at an angular velocity of 60˚/s in the isotonic and isokinetic exercise groups, and the greatest increase in muscle strength at an angular velocity of 180˚/s was detected in the isokinetic exercise group. Similarly, in our study, while a decrease in pain and an increase in functional capacity were achieved in all groups, isokinetic exercises were found to be more effective at increasing muscle strength. This significant difference was considered to have resulted from the fact that type II muscle fibers were strengthened more during the isokinetic exercises [26].

Gezginaslan et al. [27] investigated the effect of isokinetic quadriceps and hamstring strengthening exercises on the proprioception and physical function of 39 patients diagnosed with moderate osteoarthritis of the knee and a moderate risk of falls. After six weeks of treatment, they found improvement in proprioception and functionality, and a decrease in pain and risk of falls compared to pretreatment. In their study, exercise therapy was given three days a week for six weeks. In our study, treatment was applied five days a week for three weeks, and similarly, a decrease in pain and an increase in functional capacity were detected.

The effect of exercise on in vivo cartilage in the short and long term has been investigated using MRI in several studies [28-30]. In a study conducted by Maldonado et al. [31] on the effect of immobilization on knee joint cartilage in rats that had previously exercised, cartilage thickness was shown to have significantly decreased in the immobilized group, while no significant decrease was observed in the group immobilized after exercise.

A significantly larger increase was detected in the cartilage thickness of the exercise group compared with that of the control group. The authors concluded that exercise prevented degenerative changes in the femoral joint cartilage that develop in the knee joint due to immobilization [31].

In their study involving 40 patients diagnosed with osteoarthritis of the knee, Tuna et al. [18] compared the patients with a control group. Isometric quadriceps exercises and hamstring stretching exercises were applied to the patient group for three months, and the patients’ pre and post-therapy pain intensity, physical function, flexor, and extensor muscle strength, and femoral cartilage thickness were evaluated. A significant decrease was detected in the VAS scores, and a significant increase from the baseline was detected in the first and third-month checks in the isometric peak torque and total activity values at 180°/s. Although the third-month values were found to be statistically significantly higher compared with the baseline values when cartilage thickness was evaluated, no significant difference was found when the baseline values were compared with the first-month values. Tuna et al. [18] assert that a greater increase occurs in femoral cartilage thickness in the third month with isometric strengthening exercises, and report that the thickening is in direct proportion with the increase in muscle strength in this late phase [18]. In the present study, in the measurement of femoral cartilage thickness using ultrasonography, a statistically significant increase was detected in the intercondylar area and the lateral condyle measurements of the left knee in the isokinetic exercise group, and in the intercondylar area measurements of the right knee in the isometric exercise group. We believe that the post-therapy increase in cartilage thickness starts in the early phase, which may be associated with an increase in muscle strength.

Limitation

The most important limitation of this study is that the gender distribution of the patients was not similar. For this reason, gender comparisons could not be made in the study. On the other hand, we believe more comprehensive evaluations should be made in larger populations in future studies.

## Conclusions

Isometric, isotonic, and isokinetic exercises decrease pain and disability in patients with osteoarthritis of the knee. In addition, isokinetic exercises increase knee extensor and flexor muscle strength. Isokinetic exercises significantly increase femoral cartilage thickness compared with the other exercise groups. Since there are a limited number of studies comparing cartilage thickness in different exercise groups, our study can help illuminate an important gap in the literature. However, studies with longer follow-up durations and a higher number of patients are needed to draw a definite conclusion about osteoarthritis and the effect of exercise therapies on femoral cartilage thickness determined via US guidance.
